# Tissue Biomarkers in Hepatocellular Tumors: Which, When, and How

**DOI:** 10.3389/fmed.2017.00010

**Published:** 2017-02-23

**Authors:** Luca Di Tommaso, Massimo Roncalli

**Affiliations:** ^1^Pathology Unit, Humanitas Clinical and Research Center, Rozzano, Milan, Italy; ^2^Department of Biomedical Sciences, Humanitas University, Rozzano, Milan, Italy

**Keywords:** hepatocellular tumors, hepatocellular carcinoma, hepatocellular adenoma, tissue markers, predictive markers, prognostic markers

## Abstract

Few tissue markers are currently available to pathologists in the study of hepatocellular tumors. These markers should be used carefully taking into consideration not only morphology but also, and sometimes even more important, the clinical setting where the lesion to be diagnosed had developed. Glypican-3, heat shock protein 70, and glutamine synthetase (GS) are markers currently used, as a single panel, to discriminate the nature of a <2 cm hepatocellular lesion lacking radiological features of hepatocellular carcinoma (HCC) detected in a cirrhotic patient under surveillance. Their use, which can be improved by clathrin heavy chain, is mostly requested on liver biopsy. Hepatocyte paraffin 1, arginase-1, polyclonal carcinoembryonic antigen, CD10, and bile salt export pump are tissue markers used to confirm, at histology, the diagnosis of HCC made by imaging before enrollment for phase III studies on novel anti-HCC drugs. In this setting, pathologists are usually requested a conclusive diagnosis on a liver biopsy of a poorly differentiated, necrotic, enriched in stem-phenotype, carcinoma. Liver fatty acid-binding protein, serum amyloid A, C-reactive protein, prostaglandin D2 synthetase, GS, and β-catenin can be used either on biopsy or surgical specimen to classify hepatocellular adenoma into hepatocyte nuclear factor (HNF-1α) inactivated (steatotic), inflammatory, with dysregulation of sonic hedgehog and prostaglandin pathways, β-catenin mutated, and unclassified.

## Introduction

Immunohistochemistry is often defined as ancillary to morphology. Coming from the latin *ancilla*, servant, the term ancillary indicates something servant or subordinate, suggesting that immunohistochemistry should be regarded as subordinate to morphology. In other words, it is morphology that should dictate the use of immunohistochemistry. This is even more stringent in the study of hepatocellular tumors where the use of tissue biomarkers is subordinate not only to morphology but also to the clinical requests. In the daily practice, these latter are mainly restricted to the followings:

(1) histopathological diagnosis of a <2 cm hepatocellular lesion lacking radiological features of hepatocellular carcinoma (HCC) detected in a cirrhotic patient under surveillance; material: biopsy specimen; differential diagnosis: dysplastic nodules (DNs) versus HCC;
(2) histopathological diagnosis of HCC in patients with a radiological diagnosis of HCC, developed in a contest of cirrhosis, who already experienced all available treatments, to be enrolled for phase III studies on novel anti-HCC drugs; material: biopsy specimen; differential diagnosis: HCC, intrahepatic cholangiocarcinoma (iCC); mixed hepatocholangiocellular carcinoma; metastases (rare);
(3) histopathological diagnosis in a hepatocellular lesion(s) discovered in not cirrhotic liver of a patient without oncological diseases; material: biopsy (frequent in the present; less probably in the future) and surgical specimen; differential diagnosis: focal nodular hyperplasia (FNH) versus hepatocellular adenoma (HA).

In the following paragraphs, we will discuss in detail the advantages and pitfalls of biomarkers used in each of these specific clinic-pathological settings, adding some practical considerations related to personal experience.

## Small Hepatocellular Nodule in a Cirrhotic Patient

Due to radiological surveillance of cirrhotic patients, a growing number of hepatocellular nodules <2 cm are recognized: 30–40% of them are diagnosed as HCC using imaging, the remaining require histopathological diagnosis. In this setting, whose clinical, radiological, and pathological features have been described in detail elsewhere (1), a panel of biomarkers has been proposed, validated, and it is currently considered a recommended diagnostic tool. It includes glypican-3 (GPC3), heat shock protein 70 (HSP70), and glutamine synthetase (GS).

### Glypican-3

Glypican-3 is a membrane-anchored heparin sulfate proteoglycan normally expressed in fetal liver and placenta, but not in normal adult liver. Hsu et al. (2) first reported that GPC3 mRNA levels were significantly higher in HCC as compared to normal liver and non-malignant liver lesions, a result later confirmed also at protein level (3). In particular, when investigated in biopsy material, up to 60% of G1/early HCC shows immunoreactivity to GPC3, either as membrane and/or cytoplasmic staining, as well as 8% of high-grade DN (4) and occasional cirrhotic fragments (5); figures to be compared to the 85% staining observed in G2–G3 HCC.

Once excluded, the possibility of immunoreactive cirrhotic cells (usually very few or isolated in a background of a parenchyma lacking architectural atypia), a positive GPC3 staining supports a diagnosis of HGDN or early/G1 HCC. To distinguish these two lesions, staying close at the border of malignancy, GPC3-positive cells should then be quantified. Data from surgical specimen showed that 3/4 of early/G1 HCC have >10% GPC3-positive cells: if immunoreactive cells observed in the lesional biopsy largely outnumber this amount, a putative diagnosis of malignancy can be considered, pending the results of HSP70 and GS. Indeed, as will be discussed at the end of this section, the presence of a single marker positive staining will be boosted up by positive results of the other two of the panel.

### Heat Shock Protein 70

Heat shock protein 70 is an antiapoptotic protein whose overexpression allows cell survival. Chuma et al. (6), working in a subset of hepatocellular lesions with a nodule in nodule pattern of growth (outer: HGDN; inner: early HCC), showed that in the inner part HSP70 was the most abundantly upregulated gene. These results were robustly confirmed when HSP70 was tested at tissue level in surgical specimen where only 1/22 (4%) HGDN as compared to 25/32 (78%) early/G1 HCC showed a positive nucleocytoplasmic staining (7). However, when the marker was tested on liver biopsy, while retaining a 90% specificity the sensitivity decreases to 61% (4), possibly due to low immunoreactivity of archive material.

The key point in the daily use of HSP70 mainly rests in the recognition and proper interpretation of positive cells. Lesional elements are usually arranged in small cluster or in pseudoacini and should be distinguished from cords of apoptotic hepatocytes, isolated periseptal hepatocytes, and some stellate cells, which can be immunoreactive for HSP70.

### Glutamine Synthetase

Glutamine synthetase (GS) is an enzyme of nitrogen metabolism and catalyzes the conversion of glutamate and ammonia to glutamine in the liver. In normal liver, GS is expressed by one or two hepatic plates around central vein. GS is also a target of β-catenin and is upregulated when this pathway is activated. Many liver nodules, benign or malignant, show a GS overexpression: the pattern of the immunostaining (diffuse/patchy/focal, homogenous/not homogenous, strong/faint) should be evaluated carefully and interpreted according to the clinical contest to avoid misdiagnosis.

In our original study on the use of GS in the diagnosis of small hepatocellular nodules in a cirrhotic background (7), we reported a sensitivity of 69% and a specificity of 94% for a homogeneous and intense pattern of staining. We also described a heterogeneous or faint staining in six cases of HGDN, which, from a practical point of view were considered as negative. More recently, Rebouissou et al. (8) showed that diffuse, homogeneous, and strong immunoreactivity to GS (usually coupled with nuclear immunoreactivity to β-catenin) is always related to “high-activity” β-catenin mutations, while a faint or weak/heterogeneous staining is related to “weak activity” mutations. The latter characterized benign HA at low risk of malignant transformation; the former was typically observed in overt HCC as well as in lesions with borderline features between HA and HCC.

In the liver biopsy, we suggested to consider as GS positive only those lesions showing >50% strong immunoreactivity (4) and to evaluate the results together with those of GPC3 and HSP70. The study by Rebouissou et al. (8) prompts a step forward: to consider, when discovered in cirrhotic patients, as suspicious for HCC all lesions with strong and diffuse GS staining. We also suggested to consider carefully a faint GS staining when observed in liver biopsy (4): the paper of Rebouissou et al. (8) support the hypothesis that these cases harbor a “weak-activity” mutation of β-catenin with a different impact on nodule’s fate (lower risk of transformation).

### Combined Use of Markers

A positive immunostaining to GPC3, HSP70, or GS may be useful or suggestive for a diagnosis of HCC but, taken alone, perhaps with the exception of strong/diffuse GS, none of these positive results are conclusive. By contrast, the combined use of them warrants 100% specificity and 72% of sensitivity. Accordingly, international guidelines recommend the combined use of them (9) and, in our daily experience, we always face the clinical request DN versus G1/early HCC using this “panel,” i.e., using more than a single marker, approach.

**Figure 1:**
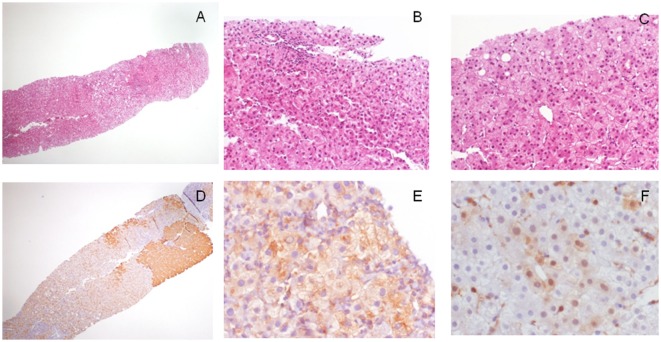
**Morphological and immunohistochemical features of a 1.7 cm hepatocellular lesion detected in a cirrhotic patient with imaging not conclusive for malignancy**. **(A)** The lesion is barely seen on the left of the biopsy (H/E, 4×); **(B)** at higher magnification, it is characterized by cell crowding and not triadal vessels (H/E, 40×); and **(C)** rare pseudoglands (H/E, 40×). Overall, morphological features are in keeping with a differential diagnosis between HGDN and early/G1 hepatocellular carcinoma (HCC). The lesion, when investigated with the diagnostic panel glypican-3 (GPC3)–heat shock protein 70 (HSP70)–glutamine synthetase (GS) showed **(D)** a strong GS staining in patchy areas (GS staining, 4×), **(E)** a focal cytoplasmic staining for GPC3 (GPC3 staining, 40×), and **(F)** the presence of scattered lesional cells arranged in cluster showing nuclear/cytoplasmic immunoreactivity for HSP70 (HSP70 immunostaining, 40×). All these findings are in keeping with a conclusive diagnosis of early/G1 HCC.

Some other makers have been proposed as useful in the diagnosis of hepatocellular nodule arising in the setting of a cirrhosis: among these, clathrin heavy chain (CHC) and EZH2 were those showing better results (10, 11). In our experience, CHC is easier to be evaluated on liver biopsy due to the fact that cytoplasmic immunoreactivity is observed, if present, only in lesional hepatocytes. Moreover, it reveals high specificity and, most important, high sensitivity: it is part of an enlarged panel represented by GPC3, HSP70, GS, and CHC we use in the diagnostic of <2 cm hepatocellular nodules discovered in cirrhotic surveillance. Figure 1 shows a typical example of a very well-differentiated hepatocellular lesion and the results of markers.

## First Histopathological Diagnosis (After Many Treatments for HCC)

According to current guidelines, the great majority of “bona fide” HCC are diagnosed according to radiological criteria, without any histological diagnosis, and treated in line with clinical criteria (9). Many of these patients experienced a liver biopsy aimed to obtain a histopathological diagnosis of HCC after many treatments (surgical, ablative, and medical) usually before enrollment in phase III clinical studies on new drug. These cases, due to their natural history and treatment as well, are poorly differentiated, partially necrotic, or even shifted toward a stem differentiation and a final diagnosis can be rendered only with the support of tissue markers. Those currently used are hepatocyte paraffin 1 (HepPar-1), arginase-1, CD10, polyclonal carcinoembryonic antigen (pCEA), GPC3, and bile salt export pump (BSEP).

### Hepatocyte Paraffin 1

Hepatocyte paraffin 1 is an antibody to carbamoyl phosphate synthetase 1, a urea cycle enzyme of mitochondria, predominantly expressed in liver and also in other organs (12). It usually shows a strong, granular, cytoplasmic immunoreactivity in normal liver, hepatoblastoma, and HCC cells. HepPar-1 has 70–84% overall sensitivity for the diagnosis of HCC, a value rising to 90% in G1 HCC; this value, however, may decrease to 20% in G3 HCC (13, 14). In addition, despite a reported overall specificity of 84%, low rates of weak positive staining have been described in gastric, lung, small intestinal, colonic, and pancreatic adenocarcinoma, as well as in melanoma, cholangiocarcinoma, ovarian, and neuroendocrine carcinoma; finally, HepPar-1 marks all tumors with hepatoid features (15). Taking into consideration all these caveats, HepPar-1, if used alone, plays a minor role in histotyping a poorly differentiated carcinoma presenting in the liver. Only cases showing strong and diffuse staining can be considered as bona fide HCC (14). By contrast, a faint positive staining, rather than helpful may, paradoxically, be an element of confusion supporting a diagnosis of HCC, which the use of a larger panel would have rule out.

### Arginase-1

Arginase isoforms 1 and 2 are involved in the hydrolysis of arginine to ornithine and urea in the urea cycle. The isoform 1 is highly expressed in the liver, while isoform 2 is expressed in the kidney and in a lesser extent in the liver. Arginase-1 is expressed at cytoplasmic and/or nuclear level. The overall sensitivity of arginase-1 in the diagnosis of HCC ranges between 84 and 96% (13, 14); in addition, even if the expression decreases as HCC grade increases, in G3 HCC sensitivity still remains between 44 and 89%, with a reported value of 54% in cases showing strong and diffuse positivity (14). The expression of arginase-1 has been reported in few cases of colon, gastric, lung, and pancreatic cancers, with a specificity for the diagnosis of HCC of 96% (13). Accordingly, the histotyping of poorly differentiated lesions of the liver may benefit of the use of arginase-1, even as single marker.

### pCEA and CD10

Polyclonal carcinoembryonic antigen stains a biliary glycoprotein similar to CEA expressed by the bile canaliculi and ducts. pCEA shows a peculiar pattern of staining in normal hepatocytes represented by a faint, spider-like, reaction in the canalicular pole of the hepatocyte; this so-called “canalicular pattern” is extremely characteristic and highly specific for HCC. Only few cells of HCC, in rare cases, may show a cytoplasmic staining; this latter, by contrast, is typical of metastatic adenocarcinoma and iCC.

CD10 is a membrane metalloendopeptidase, which cuts the amino group of hydrophopic residues. It is normally expressed in cytoplasm/membrane of many adenocarcinomas, while in normal hepatocytes and HCC, it shows the same “canalicular pattern” observed with pCEA. Despite an almost 100% specificity, the “canalicular pattern” is affected by a low sensitivity for both pCEA (45–81%) and CD10 (50–74%); interestingly enough, these values rise up to 78 and 67%, respectively, in G3 HCC.

### Glypican-3

Glypican-3 expression has been reported in melanomas, extragonadal germ cell tumors, squamous cell carcinoma of the lung, squamous- and adenocarcinoma of the esophagum, ovarian tumor, and, most important, in up to 14% of GI and pancreatic liver metastasis (6), with an overall specificity for HCC of 86% (16). Thus, despite GPC3 has an 89% sensitivity (16) for G2–G3 HCC, the use of GPC3 alone to establish the exact histogenesis of a liver tumor should be evaluated carefully.

### Bile Salt Export Pump

Bile salt export pump is an ATP-binding cassette transporter expressed almost exclusively in the canalicular part of hepatocytes. In a recent study, Lagana et al. (17) investigated the efficiency of BSEP to identify HCC against all the other markers mentioned above. BSEP showed a convincing canalicular staining in 76% of cases and cytoplasmic/dot in the remaining. BSEP, CD10, and pCEA showed 100% specificity as compared to 97% of GPC3 and HepPar-1 and 94% of arginase. BSEP and arginase showed the highest sensitivity (90%), a value progressively decreasing with HepPar-1 (90%), pCEA (81%), CD10 (74%), and GPC3 (54%).

Table 1 illustrates the performances of the each of these histotype markers when used alone. It should be observed that only Radwan and Ahmed (13) investigated the performance of two of these markers in combination (arginase and HepPar-1) and reported 100% specificity coupled with 70% sensitivity. According to our experience with biomarkers, this “panel” approach warrants optimal results and should be encouraged also in this field.

**Table 1:** **Sensitivity of markers used to prove hepatocellular carcinoma (HCC) nature of a liver lesion**.

Marker	All HCC, sensitivity (%)	G3 HCC, sensitivity (%)
Hepatocyte paraffin 1	70–84	22–78
Arginase	84–96	44–89
Polyclonal carcinoembryonic antigen	45–81	78
CD10	50–74	67
Glypican-3	54	67
Bile salt export pump	90	78

## An Hepatocellular Nodule in Otherwise Unremarkable Liver

The detection of a, usually single, liver lesion, in a patient without known oncological disease and cirrhosis, restricts the differential diagnosis to two hepatocellular lesions: FNH and HA. Translational studies, mainly coming from a French network of researchers, recently defined a robust classification of HA with strict correlation between molecular features, morphology, clinical aspects, and imaging (18–21). In addition, these studies provided to the pathologists a number of tissue markers, namely, liver fatty acid-binding protein (LFABP), serum amyloid A (SAA), PCR, as well as GS and β-catenin, and more recently PTGDS (21), which allows to recognize these lesions on tissue specimen. The latter can be represented by a liver biopsy, a frequent event in the current daily practice; recent EASL guidelines (22) however did not contemplate the possibility of a biopsy in the flowchart of HA (which is included by contrast in the diagnosis of FNH), thus possibly restricting the use of these markers to surgical specimen. A detailed, pathology oriented, review of these lesions has been reported elsewhere (23).

### Liver Fatty Acid-Binding Protein

Liver fatty acid-binding protein is downregulated in HNF-1α inactivated HA. The latter are characterized by a steatotic morphology, hence the common name of steatotic HA (s-HA). HNF-1α inactivated HA are usually easily recognized at imaging (22) and has a low potential of malignant transformation; in the rare cases where an histopathological diagnosis is requested, it rests in the identification of a steatotic nodule with negative LFABP staining merging into an otherwise normal parenchyma with a positive cytoplasmic LFABP staining. Recently, Cho et al. (24) reported that a negative LFABP can also be observed in HCC; thus, a negative staining without adequate morphology is not diagnostic *per se* of an s-HA.

### SAA/C-Reactive Protein (CRP)

Serum amyloid A associated protein and CRP are upregulated in inflammatory HA (i-HA) as a result of activating mutation along JAK–STAT pathway. SAA is expressed at cytoplasmic level by lesional hepatocytes as well as in some hepatocytes surrounding any liver mass and in FNH. PCR shows a similar strong, cytoplasmic staining in lesional hepatocytes, coupled with a larger amount of patchy immunoreactivity in non-lesional cells. The combined use of SAA and CPR avoid false positive results.

### Glutamine Synthetase

HNF-1α inactivated HA, unclassified HA (HA, nos), and most i-HA are negative to GS staining; a faint/focal immunoreactivity to GS is observed in exon 7/8 β-catenin mutated HA (β-HA); a strong/diffuse positivity is observed in exon 3 β-catenin mutated HA (8). A strong immunoreactivity, however, is observed also in FNH: in this lesion, however, the immunoreactivity is not diffuse rather “map-like,” the latter describing the presence of large, anastomosing group of positive hepatocytes, surrounding vessels, and sparring the hepatocytes close to fibrotic bands.

**Figure 2:**
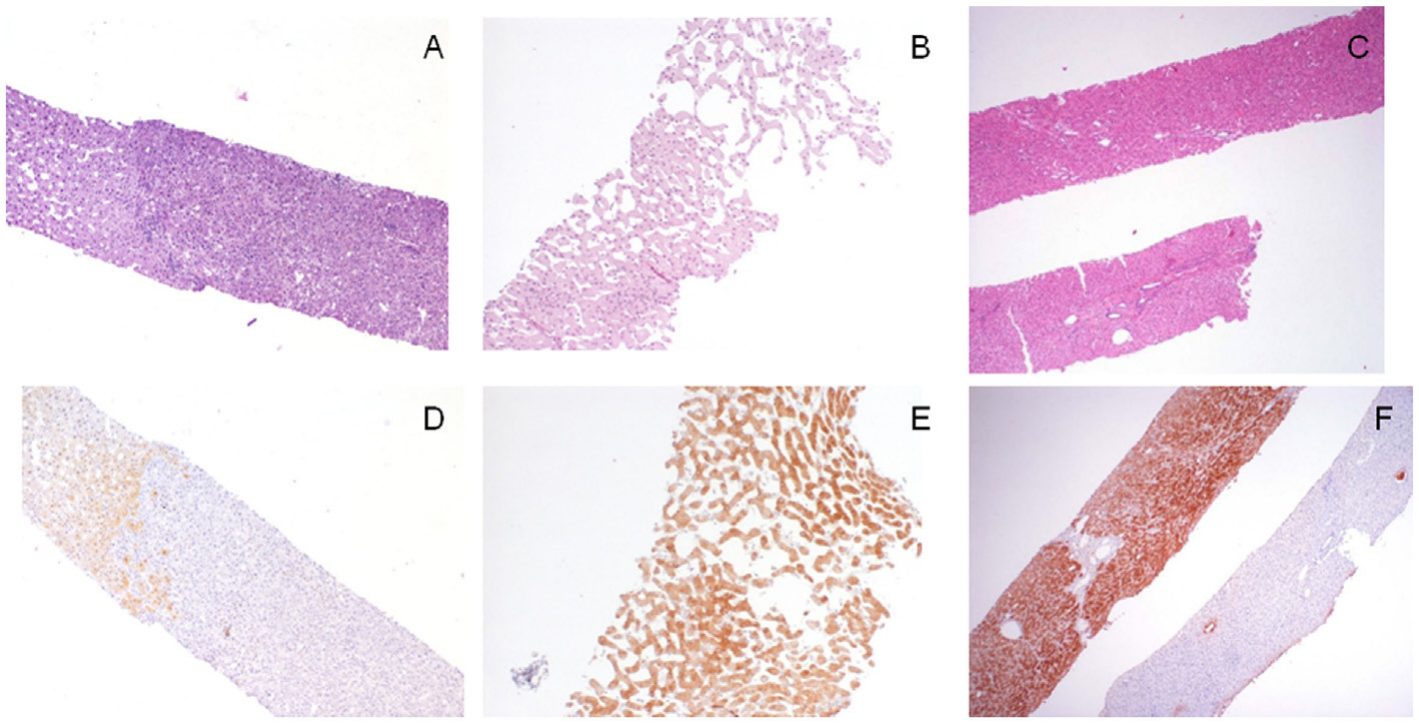
**Morpho-phenotypical correlations in the hepatocellular adenomas (HAs)**. **(A,D)** This hepatocellular lesion [**(A)**, H/E, 4×, right part of the biopsy] is negative to liver fatty acid-binding protein (LFABP) staining [**(D)**, LFABP staining, 4×] findings consistent with a final diagnosis of HNF-1α inactivated, steatotic, HA; LFABP cytoplasmic positivity is always observed in normal parenchyma. **(B,E)** This hepatocellular lesion characterized by ectatic sinusoids [**(B)**, H/E, 10×] shows diffuse cytoplasmic immunoreactivity to serum amyloid A (SAA) [**(E)**, SAA immunostaining, 10×], which is diagnostic for an inflammatory (telangiectatic) HA. **(C,F)** The lesional biopsy [**(C)**, H/E, 4×, upper fragment] obtained from a 3 cm hepatcellular nodule in an 18-year-old man shows a strong and diffuse immunoreactivity to glutamine synthetase (GS); this finding is consistent with a diagnosis of an atypical (β-catenin mutated) adenoma that was further confirmed by nuclear immunoreactivity to β-catenin not shown; note for comparison in the lower fragment the normal GS staining in the perivenular areas.

In the setting of a liver biopsy obtained from a hepatocellular nodule in unremarkable liver, GS can be extremely useful to pathologists. Thus, a lesion characterized by the presence of fibrous tissue, suggestive but not conclusive for FNH on H/E, can be diagnosed easily if showing a “map-like” GS staining; the worrisome findings of hepatocytes with increased N/C ratio arranged in acinar structure can be better set as an atypical HA if supported by a diffuse/strong GS staining. Figure 2 shows some examples of LFABP, SAA, and GS staining in different varieties of HA.

**Figure 3:**
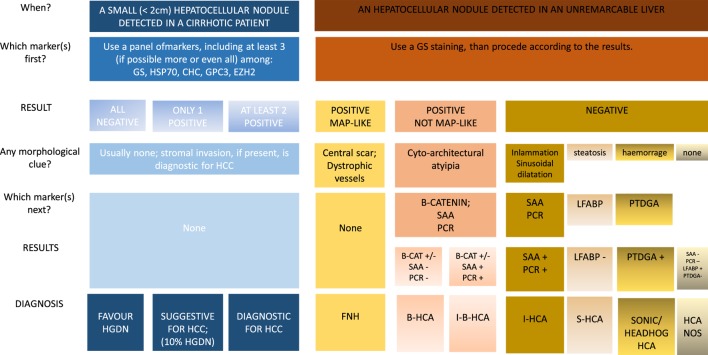
**Algorithmic approach to the use of tissue biomarkers in the clinical setting of small hepatocellular nodules detected in cirrhotic patients under surveillance and in hepatocellular nodules detected in unremarkable liver**.

Finally, it should be observed that some HAs merge into extremely well-differentiated HCC. In these lesions, the differential diagnosis between a benign and a malignant lesion can be extremely difficult especially on liver biopsy. From a practical point of view, two different approaches have been suggested to these lesions. Bedossa et al. (25) suggested to consider these lesions as of uncertain malignant potential while Nguyen et al. (26) prompted to use HSP70 and GS to distinguish HCC from atypical HA. An algorithmic approach to the use of tissue markers in the diagnosis of hepatocellular nodule in a cirrhotic or unremarkable liver, is proposed in Figure 3.

## Prognostic and Predictive Tissue Biomarkers: Where are We Going?

Several other biomarkers have been investigated in HCC. Some have been reported as prognostic biomarkers, possibly related to a stem cell phenotype. For some of these markers, osteopontin is an example, further studies failed to confirm the prognostic role at tissue level; for others, like CK19, data on tissue expression are robust but their use in the daily practice has never been endorsed by international guidelines. Other markers have been suggested as able to discriminate patients eligible to a certain treatment (predictive markers). Among these, VEGF and CAIX have been described as possible predictive tissue biomarkers of TACE resistance (27). More recently, the evaluation of c-MET at tissue level has been related to the response to Tivantinib (28) while the use of Nivolumab turned out to be not predictable by PDL1 at tissue level (29).

These frustrating results should not discourage the study of novel biomarkers and their translation at tissue level into prognostic and predictive indicators. In the meanwhile, the best approach seems that suggested by Tsujikawa et al. (30): to use all the available tissue markers to separate HCC into subgroups reflecting tumor aggressiveness. This approach, in line with the work done with breast cancer, can be extremely useful in the daily practice.

## Author Contributions

LDT: collected the data, general organization of the project, wrote the paper, edited the manuscript, and collected pictures; MR: general review.

## Conflict of Interest Statement

The authors declare that the research was conducted in the absence of any commercial or financial relationships that could be construed as a potential conflict of interest.
